# Impact of intracranial pressure and related parameters on prognosis in patients undergoing surgery for ruptured intracranial aneurysms

**DOI:** 10.1007/s10143-025-04016-9

**Published:** 2025-12-20

**Authors:** Tao Wang, Yan Xiao, Quan Liao, Feiyifan Wang, Yuanding Jiang, Shenghua Cui, Ping Chen, Yajie Huo, Jing Shu, Wentao Fan, Chenzhuo Li, Xiaoling Deng, Peng Li, Zhoucheng Lu, Yonghong Duan, Richu Liang

**Affiliations:** 1 Department of Neurosurgery, The Second Affiliated Hospital, Hengyang Medical School, University of South China, Hengyang, Hunan China; 2 The Second Xiangya Hospital of Central South University, Changsha, Hunan China; 3 Department of Intensive Care Medicine, The Second Affiliated Hospital, Hengyang Medical School, University of South China, Hengyang, Hunan China

**Keywords:** Aneurysmal subarachnoid hemorrhage, Intracranial pressure monitoring, Decompressive craniectomy, CPPopt, Prognosis

## Abstract

**Objective:**

This study aimed to clarify the prognostic significance of intracranial pressure (ICP) monitoring in patients with aneurysmal subarachnoid hemorrhage (aSAH) who underwent surgical treatment. Specifically, we analyzed clinical demographic data and ICP-related parameters (e.g., optimal cerebral perfusion pressure [CPPopt], ΔCPP) to identify key prognostic indicators, with the ultimate goal of providing evidence to guide and optimize postoperative management strategies for this patient population.

**Design:**

Retrospective analysis of prospectively collected data.

**Setting:**

Neurosciences critical care unit of a university hospital.

**Patients:**

A prospective cohort of 15 aSAH patients undergoing multiparameter monitoring (April 2024–January 2025) and a retrospective cohort of 45 surgically treated aSAH patients (2022–2024) were included.

**Interventions:**

None.

**Measurements and main results:**

Demographic data, ICP monitoring results, and Glasgow Outcome Scale (GOS) scores were collected from all patients at one month postoperatively. The absolute difference between the optimal cerebral perfusion pressure (CPPopt) and the 15-min mean cerebral perfusion pressure (CPP) was defined as ΔCPP.​

Patients were stratified into two groups based on their GOS scores: the favorable prognosis group (GOS 4–5) and the poor prognosis group (GOS 1–3). Subsequently, statistical analysis was performed to explore the associations between prognostic outcomes and both demographic data (e.g., gender, age, surgical approaches) and monitoring parameters (e.g., pressure reactivity index [PRx], pressure reactivity slope [RAP], ICP, ΔCPP).

A retrospective cohort of 52 patients with aSAH — treated at the Second Hospital of the University of South China between 2022 and 2024 — was matched with a prospective cohort at a 1:3 ratio using propensity score matching (conducted via R software). After matching, 45 patients from the prospective cohort were retained as controls. Baseline characteristics were well-balanced between the retrospective (test) group and the prospective (control) group, and the distributions of prognostic outcomes were compared between the two groups.

Elevated initial ICP was a strong predictor of poor prognosis (41.33 ± 10.23 vs. 25.89 ± 10.42 mm Hg, p = 0.014). In patients with ICP ≥ 25 mm Hg, decompressive craniectomy (DC) was associated with a trend toward improved outcomes (Relative Risk [RR] ≈4.8, p = 0.08).​

The poor-outcome group exhibited a greater mean ΔCPP compared with the favorable-outcome group (12.88 vs. 10.61, p = 0.033). Hypoperfusion (i.e., cerebral perfusion pressure [CPP] < optimal CPP [CPPopt]) was significantly associated with adverse outcomes (p = 0.038). In contrast, hyperperfusion (i.e., CPP > CPPopt) was not significantly associated with harm (p = 0.084).​

Furthermore, patients who underwent ICP monitoring had a higher favorable prognosis rate than those without monitoring (60% vs. 31.1%, p = 0.046).

**Conclusions:**

First, in the clinical management of aSAH patients, ICP monitoring plays a pivotal role: it not only facilitates surgical decision-making (e.g., selection of decompressive craniectomy) but also optimizes postoperative hemodynamic management strategies tailored to individual patients.​

Second, two key factors were identified to predict poor prognosis in aSAH patients: one is the deviation from postoperative CPPopt, among which hypoperfusion is a particularly impactful subtype; the other is elevated initial ICP.​

Furthermore, this study confirmed that management guided by multiparameter monitoring significantly improves short-term clinical outcomes in aSAH patients, providing practical evidence for the optimized clinical management of this patient population.

## Introduction

Aneurysmal subarachnoid hemorrhage (aSAH) is a common neurosurgical emergency associated with high mortality. The global incidence of aSAH is reported to be 10.61 per 100,000 person—years, and its disability and mortality rates remain extremely high [1]. Despite significant advances in clinical diagnosis and treatment over recent years, the prognosis of patients with aSAH remains unsatisfactory.Neurological impairment in aSAH patients is primarily attributed to hypoperfusion induced by intracranial hypertension (ICH) and cerebral vasospasm. Furthermore, their poor prognosis is closely linked to key complications, including elevated intracranial pressure (ICP), cerebral vasospasm, and delayed cerebral ischemia [2, 3].

Current clinical management of ICP and cerebral perfusion pressure (CPP) in patients with aSAH is guided by traumatic brain injury (TBI)—related clinical guidelines. These guidelines advocate for maintaining CPP within the range of 60–70 mm Hg and recommend initiating ICP intervention when ICP exceeds the threshold of 20–22 mm Hg [4].Notably, clinical management strategies for aSAH patients are often extrapolated from TBI guidelines, given the lack of aSAH—specific consensus. However, these identical targets (for CPP) and intervention thresholds (for ICP) may not be universally applicable to these two distinct disease entities, as aSAH and TBI differ in pathological mechanisms, disease progression, and cerebral hemodynamic characteristics [5].

In recent years, the concept of optimal cerebral perfusion pressure (CPPopt) has gained growing recognition in neurocritical care, reflecting its potential clinical relevance for guiding cerebrovascular management in critically ill neurosurgical patients. Notably, the Pressure Reactivity Index (PRx)—a validated parameter for assessing cerebrovascular autoregulatory capacity [6]—is calculated as the dynamic correlation between ICP and mean arterial pressure (MAP) waveforms, a measurement that directly links systemic hemodynamics to cerebral vascular responsiveness.

Effective cerebrovascular autoregulation sustains relative stability of MAP and ICP, which in turn results in low correlation between these waveforms and correspondingly small PRx values. In contrast, when autoregulatory function is impaired (e.g., in cerebral vasospasm or cerebral ischemia—common complications in neurosurgical practice), the correlation between MAP and ICP waveforms increases, leading to elevated PRx values that signal compromised cerebral hemodynamic control. By serial monitoring of PRx across a spectrum of CPP levels, a U-shaped PRx-CPP relationship can be derived; the nadir of this curve—corresponding to the minimum PRx value—defines CPPopt. This parameter represents a dynamic, autoregulation-driven target, specifically denoting the CPP at which cerebrovascular autoregulation operates with maximal efficiency [7, 8].

In TBI—a core focus of neurosurgical critical care—studies have demonstrated that the absolute difference between measured CPP and CPPopt correlates with adverse clinical outcomes in affected patients [9]. Furthermore, TBI patients whose measured CPP closely approximates CPPopt exhibit significantly better prognostic trajectories compared to those with substantial deviations from this optimal threshold [10, 11]. Extending this concept to aSAH—another key neurosurgical condition—CPPopt holds promise as a prognostic marker; however, its predictive utility remains incompletely characterized. This knowledge gap stems from a paucity of high-quality studies and the lack of validation in large, heterogeneous aSAH cohorts. Additionally, the clinical efficacy of PRx-guided CPP optimization strategies—an intervention relevant to neurosurgical care pathways—requires further rigorous investigation.​

Thus, quantifying the deviation of measured CPP from CPPopt and elucidating its prognostic significance in neurosurgical populations (e.g., TBI, aSAH) represent critical priorities for future research, with potential to refine hemodynamic management protocols in neurocritical care.

Traditional ICP monitoring focuses only on ICP itself, but a single parameter is difficult to fully reflect the complex changes in cerebral hemodynamics.Recent advances in multimodal neuromonitoring (MMN) integrate critical parameters – ICP, CPP, brain tissue oxygenation (PbtO₂), and PRx – enabling comprehensive assessment of post-injury cerebrovascular dynamics and real-time clinical decision support [12, 13]. Multimodal neuromonitoring (ICP, PRx, CPPopt) enables early detection of pathophysiological changes post-brain injury, guiding targeted interventions to optimize outcomes in aSAH (aSAH). Emerging real-time autoregulation metrics (RAP, PRx) and CPPopt-driven protocols are expected to provide more effective clinical tools in predicting and intervening in cerebral blood flow abnormalities (e.g., DCIs), helping to improve patient outcomes and quality of life.

This study analyzed clinical data and ICP parameters in patients with aSAH to evaluate their prognostic significance in surgically treated ruptured intracranial aneurysms, thereby exploring the clinical utility of Multiparameter monitoring-guided postoperative management.

## Methods

### Patients

This mixed prospective-retrospective study enrolled patients with aSAH treated at the Department of Neurosurgery, The Second Hospital, University of South China. The prospective cohort comprised consecutive patients admitted between April 2024 and January 2025, while the retrospective cohort included cases treated between 2022 and 2024. All patients were definitively diagnosed via CTA, MRA, or DSA.

Prospective cohort inclusion criteria: Emergency hospital admission within ≤ 3 days after aSAH ictus.Confirmed diagnosis of aSAH (via CTA or DSA) without concurrent vascular malformations.Underwent surgical intervention for ruptured intracranial aneurysm (either surgical clipping or endovascular coiling) with intraoperative placement of both a ventricular drainage catheter and an ICP monitoring probe.

Inclusion criteria of the retrospective cohort: Emergency admission ≤ 3 days post-ictus.Confirmed aSAH without vascular malformations (CTA/DSA). Surgical intervention (clipping/coiling) with ventricular drainage.

Exclusion criteria: Subarachnoid hemorrhage (SAH) of non-aneurysmal etiology (e.g., traumatic SAH). Severe preexisting comorbidities that could confound outcome assessment (e.g., congenital coagulation disorders, end-stage organ failure [heart, liver, or kidney]). History of prior cerebral hemorrhage (including intracerebral, intraventricular, or subarachnoid hemorrhage). Loss to follow-up during the predefined study follow-up period, or incomplete clinical, radiological, or monitoring data (insufficient for outcome or parameter analysis).

Prospective Cohort: A total of 18 patients were initially screened for the prospective cohort. Of these, 3 were excluded due to the following reasons: 1 case of technical failure leading to incomplete monitoring data, 1 case of intraoperative ICP probe misplacement (rendering ICP measurements unreliable), and 1 case of voluntary withdrawal of care by the patient’s family. The final prospective cohort comprised 15 patients with complete continuous ICP monitoring data.

Retrospective Cohort: The retrospective cohort was initially composed of 52 patients identified via strict application of the aforementioned inclusion and exclusion criteria. To minimize selection bias between cohorts, propensity score matching (PSM) was performed, with the 15 patients from the prospective cohort serving as the reference group.Matching variables included key baseline clinical and radiological characteristics: age, gender, preoperative Glasgow Coma Scale (GCS) score, Hunt-Hess grade, modified Fisher grade, and surgical intervention type (clipping vs. coiling)—all factors known to influence aSAH outcomes and thus potential confounders. PSM was conducted at a 1:3 ratio (prospective:retrospective) using R software. This process yielded 45 successfully matched patients in the retrospective cohort, ensuring balanced baseline characteristics between the two cohorts.

### Therapeutic options for prospective cohorts

Upon admission, all suspected aSAH patients underwent urgent cranial non-contrast CT (to identify SAH) and CTA (to confirm aSAH and localize the ruptured aneurysm).​Concurrently, standardized assessments were conducted to stratify severity: GCS, Hunt-Hess grade, and modified Fisher grade.Vital signs (systolic/diastolic blood pressure, heart rate, respiratory rate, peripheral oxygen saturation) and neurological status (mental state, pupillary reflexes) were monitored continuously to detect early neurological deterioration or hemodynamic instability. Osmotic agents (e.g., mannitol) were used for ICP control in patients with elevated ICP; agitated patients received sedation (e.g., midazolam) and analgesia (e.g., remifentanil) to maintain stability and optimize neurological assessment.

Surgical Intervention: Definitive treatment (surgical clipping or endovascular coiling) was prioritized based on aneurysm characteristics (location, size), systemic status, and family economic condition. Preoperatively, a ventricular ICP probe (Spiegelberg® monitor) was placed via frontal horn puncture for real-time ICP monitoring during surgery and ICU admission.

Postoperative Management: ICP Monitoring: Continuous data acquisition via NeumaticDCR® system (Shanghai Haoju). Sedation/analgesia to minimize ICP fluctuations.CSF drainage via ventricular catheter for refractory ICP elevation. Escalated osmotic therapy (mannitol/hypertonic saline). Repeat head CT and neurosurgical consultation if ICP remained uncontrolled, with consideration of reoperation. All patients received nimodipine for vasospasm prophylaxis, alongside standard supportive measures: fluid-electrolyte balance, nutritional support, infection control, and thromboprophylaxis. Treatment for all patients was adjusted based on real-time CPP and the current CPPopt. The therapeutic target was set to maintain CPP within CPPopt ± 10 mmHg. This target was achieved by adjusting blood pressure (using vasoactive agents) and/or intracranial pressure (using sedation, cerebrospinal fluid drainage, hyperventilation, etc.). The therapeutic target was adjusted accordingly after each update of the CPPopt value.

### Data collection and organization

Case data: gender, age, surgical procedure, initial ICP, whether or not decompressive craniectomy, Hunt-Hess grading and modified Fisher grading at admission were collected through the electronic medical record system.

ICP monitoring data: The ICP monitor was zeroed intraoperatively for real-time pressure measurement, and the data processing tool (NeumaticDCR data acquisition system, Shanghai Haoju Company) was connected immediately after admission to the ICU to monitor and record the ICP, CPP, RAP, and PRx according to the standard procedure, and the raw data collected by the NeumaticDCR data acquisition system was used to reject the abnormal values. The raw data collected by the NeumaticDCR data acquisition system were pre-processed to obtain ICP, CPP, PRx, CPPopt and other ICP-related parameters. The Neumatic DCR data acquisition system automatically collects CPPopt (CPP-PRx curve) based on real-time CPP-PRx curves. By monitoring PRx changes at different CPP levels, a U-shaped curve of PRx relative to CPP is obtained. The lowest point of this U-shaped curve—where PRx reaches its minimum value—typically corresponds to the optimal state of cerebral blood flow regulation. The CPP value at this point is considered the CPPopt. The system records real-time CPP and the corresponding CPPopt value every 15 min.

Outcome data: The Glasgow prognostic score (GOS score) was obtained by telephone follow-up or outpatient review at 1 month after surgery.

Data organization: For data organization, patients were first stratified into two prognostic groups based on 1-month GOS scores: the poor prognosis group (GOS 1–3) and the favorable prognosis group (GOS 4–5). Baseline and clinical variables were then compared between these two prognostic groups, including demographics (age, sex), surgical factors (procedure type, decompressive craniectomy), and disease severity indicators (initial ICP, Hunt-Hess grade categorized as 1–2 vs. 3–5, and modified Fisher grade categorized as I–II vs. III–IV). Next, the calculation of ΔCPP (the absolute difference between CPPopt and 15-min averaged CPP values) was defined, with the formula for mean ΔCPP specified as mean ΔCPP = (15/T) × ∑|CPPopt—CPP| (where T represents total monitoring duration in minutes, and the absolute value ensures consistent measurement of difference magnitude). Finally, subgroup analysis of ΔCPP was conducted, with ΔCPP evaluated in two subgroups: one where CPP was greater than CPPopt (hyperperfusion-related ΔCPP) and another where CPP was less than CPPopt (hypoperfusion-related ΔCPP).

### Statistical analyses

Continuous Variables (e.g., age, initial ICP, ΔCPP):Normality assessed via Shapiro–Wilk test. Normally distributed: Expressed as mean ± SD; compared using independent t-tests. Non-normal: Analyzed with nonparametric tests (e.g., Mann–Whitney U). Categorical Variables: Expressed as counts (percentages). Intergroup comparisons: Chi-square test or Fisher’s exact test (if expected cell frequency < 5). Then, variables with statistical significance in the univariate analysis were included in the multivariate binary logistic regression analysis.

### Ethical reviews

This study was conducted in accordance with the ethical principles of the Declaration of Helsinki. The study was reviewed and approved by the Ethics Committee of the Second Hospital, University of South China (Authorization No.:2024049),and has been registered with the China Clinical Trial Registry (https://www.chictr.org.cn/index.html. registration number: ChiCTR2500097852). Written informed consent was obtained from all participants or their legally authorized representatives before participation.

## Results

### Prospective cohort characteristics

The final prospective cohort included 15 patients (male: 40%, female: 60%; mean age: 56.8 ± 9.7 years). All underwent ventricular ICP probe placement and definitive treatment: 66.7% (n = 10) received surgical clipping, while 33.3% (n = 5) underwent interventional embolization. Disease severity was stratified as follows: Hunt-Hess grade: 60% (n = 9) low-grade (1–3), 40% (n = 6) high-grade (4–5).Modified Fisher grade: 13.3% (n = 2) low-grade (I–II), 86.7% (n = 13) high-grade (III–IV). Decompressive craniectomy was performed in 33.3% (n = 5), all with initial ICP ≥ 25 mm Hg. Mean initial ICP was 32.07 ± 12.68 mm Hg (range: 15–60 mm Hg). At 1-month follow-up, 60% (n = 9) achieved favorable outcomes (GOS 4–5), while 40% (n = 6) had poor outcomes (GOS 1–3) (Table 1).

**Table 1:** Demographic and Clinical Characteristics of the Prospective Cohort

Variable		statistical results
Sex	Male	6(40%)
	Female	9 (60%)
Age(years)		56.8 ± 9.7(Range 40—73)
Hunt-Hess Grading	1–3	9 (60%)
	4–5	6 (40%)
Improved Fisher Grading	1–2	2 (13.3%)
	3–4	13 (86.7%)
Surgical methods	Cranial aneurysm clamping	10 (66.7%)
	Interventional embolization	5 (33.3%)
Decompressive Craniectomy(DC)	Yes	5 (33.3%)
	No	10 (66.7%)
Initial ICP(mmHg)		32.07 ± 12.68(Range 15—60)
PRx		0.01 ± 0.66(Range -0.16—0.11)
RAP		0.56 ± 0.23(Range 0.22—0.86)
GOS	Favorable prognosis (4–5)	9 (60%)
	Poor prognosis(1–3)	6 (40%)

### Comparison of each parameter in the prospective cohort's Favorable prognosis and poor prognosis groups

Patients in the prospective cohort were categorized into two groups according to the GOS prognostic scoring scale as good prognostic group (GOS = 4–5 points) and poor prognostic group (GOS = 1–3 points), and the relationship between variables such as age, gender, surgical approach, whether or not to decompressive craniectomy, initial ICP, Hunt-Hess grade, modified Fisher grade, PRx, RAP, and ΔCPP, etc., and the prognosis was compared, and a univariate analysis was performed. Meanwhile, the normality of continuous numerical type variables (age, initial ICP, PRx, RAP, mean ΔCPP deviating from CPPopt, mean ΔCPP higher than CPPopt, mean ΔCPP lower than CPPopt) was assessed, and Shapiro–Wilk tests showed p-values > 0.05, which were consistent with a normal distribution. Therefore, they were subjected to independent samples t-test and Fisher exact test for other categorical variables.

Non-Significant Variables: Gender, age, surgical method, Hunt-Hess grade, modified Fisher grade, PRx, and RAP showed no significant intergroup differences (p > 0.05).

ΔCPP and Prognosis: Overall ΔCPP: Poor-outcome patients exhibited significantly higher mean ΔCPP (12.88 vs. 10.61, p = 0.033), indicating CPP deviation from CPPopt as a risk factor for adverse outcomes. Hypoperfusion (CPP < CPPopt): Strongly associated with poor prognosis (p = 0.038). Hyperperfusion (CPP > CPPopt): Trend toward harm (ΔCPP: 10.05 vs. 7.05, p = 0.084), nearing statistical significance.Initial ICP Impact: Poor-outcome group had higher initial ICP (41.33 ± 10.23 vs. 25.89 ± 10.42 mm Hg, p = 0.014), supporting elevated ICP as a poor prognostic marker (Table 2).

**Table 2:** Univariate analysis of variables and prognosis

		Favorable prognosis (n = 9)	Poor prognosis(n = 6)	Statistical quantity	*p*
Sex	Male	5(55.6%)	1(16.7%)	Fisher’s exact test	0.287
Age		55.0 ± 10.67	59.5 ± 8.2	−8.72	0.399
Cranial aneurysm clamping		6(66.7%)	4(66.7%)	Fisher’s exact test	> 0.999
DC		4(44.4%)	1(16.7%)	Fisher’s exact test	0.580
Hunt-Hess		4(44.4%)	2(33.3%)	Fisher’s exact test	0.608
Improved Fisher Grading		8(88.9%)	5(83.3%)	Fisher’s exact test	> 0.999
Initial ICP(mmHg)		25.89 ± 10.42	41.33 ± 10.23	−2.832	0.014
PRx		0.023 ± 0.055	−0.018 ± 0.077	1.228	0.241
RAP		0.52 ± 0.22	0.62 ± 0.24	−0.813	0.431
Average△CPP		10.61 ± 1.65	12.88 ± 2.02	−2.387	0.033
Average△CPP(greater than optimal)		7.05 ± 2.23	10.05 ± 4.02	1.868	0.084
Average△CPP(less than optimal)		11.38 ± 1.94	13.63 ± 1.68	2.311	0.038

In the univariate analysis of the aforementioned study, statistically significant variables included initial ICP, mean ΔCPP deviating from CPPopt, and mean ΔCPP below CPPopt. Incorporating these three variables into a multivariate binary logistic regression analysis revealed that initial ICP (P = 0.181, OR = 1.140, 95% CI = 0.941–1.381), mean ΔCPP deviating from CPPopt (P = 0.934, OR = 1.115, 95% CI = 0.086–14.506), and mean ΔCPP below CPPopt (P = 0.878, OR = 1.231, 95% CI = 0.086–17.537) all yielded P values > 0.05, indicating no statistical significance.

Decompressive Craniectomy (DC): In patients with ICP ≥ 25 mm Hg (n = 11), DC trended toward improved outcomes (favorable prognosis: 80% vs. 16.7% non-DC; RR≈4.8, p = 0.08). While statistically inconclusive, this suggests potential clinical relevance (Table 3).

**Table 3:** Univariate analysis of decompressive craniectomy and prognosis in patients with ICP ≥ 25 mmHg

ICP ≥ 25 mmHg		Favorable prognosis(n = 5)	Poor prognosis(n = 6)	statistical quantity	*p*
DC	Yes	4(80%)	1(16.7%)	Fisher’s exact test	0.08
	No	1 (20%)	5 (83.3%)	Fisher’s exact test	

Our prior prospective analysis identified postoperative ΔCPP (deviation from CPPopt) and elevated initial ICP as critical determinants of outcomes in aSAH (aSAH). To evaluate the clinical utility of multimodal neuromonitoring, we compared short-term prognosis between: Test group: Prospective cohort with multiparameter-guided management (ICP, CPPopt, ΔCPP).Control group: Retrospective cohort managed without advanced monitoring.

### Control cohort assembly

A retrospective cohort of 52 aSAH patients (treated at the Second Hospital, University of South China, 2022–2024) underwent 1:3 propensity score matching (R software) with the prospective cohort. After matching, 45 patients were retained as controls. Baseline characteristics—including age, sex, surgical method, Hunt-Hess grade, modified Fisher grade, decompressive craniectomy status, and GCS score—showed no significant intergroup differences (p > 0.05, Table 4), confirming balanced cohorts for comparative analysis.

**Table 4:** Results of propensity matching between test group and control group

		Control group	Test group	*p*
n		45	15	
Sex(%)	Male	20(44.4)	6(40.0)	> 0.999
	Female	25 (55.6)	9 (60.0)	
Age(%)	< 60	27 (60.0)	8 (53.3)	0.88
	≥ 60	18 (40.0)	7 (46.7)	
Surgical methods (%)	Cranial aneurysm clamping	33 (73.3)	10 (66.7)	0.869
	Interventional embolization	12 (26.7)	5 (33.3)	
Hunt-hess(%)	2	12 (26.7)	3 (20.0)	0.868
	3	13 (28.9)	6 (40.0)	
	4	16 (35.6)	5 (33.3)	
	5	4 (8.9)	1 (6.7)	
Improved Fisher Grading (%)	2	9 (20.0)	2 (13.3)	0.648
	3	10 (22.2)	5 (33.3)	
	4	26 (57.8)	8 (53.3)	
DC(%)	No	27 (60.0)	10 (66.7)	0.878
	Yes	18 (40.0)	5 (33.3)	
GCS (%)	≤ 8	20 (44.4)	6 (40.0)	> 0.999
	> 8	25 (55.6)	9 (60.0)	

### Comparison of prognosis between the test group and the control group

With balanced baseline characteristics between cohorts (p > 0.05), comparative analysis revealed a significantly higher rate of favorable prognosis (GOS 4–5) in the multimodal monitoring group (60%) versus the control group (31.1%) (Χ^2^ = 3.972,p = 0.046, Table 5).

**Table 5:** Comparison of prognostic distribution between the test group and control group

	Favorable prognosis	Poor prognosis	Percentage with favorable prognosis	Χ^2^	*p*
Control group (n = 45)	14	31	31.1%	3.972	0.046
Test group (n = 15)	9	6	60%		

## Discussion

aSAH represents a major global health burden as the third most prevalent cerebrovascular event after ischemic stroke and hypertensive hemorrhage. While advancements in diagnostic and therapeutic techniques have improved outcomes, the prognosis remains guarded, with many survivors facing persistent cognitive/motor deficits or mortality. Using univariate analysis, this study assessed the association between ICP-related parameters and clinical outcomes in aSAH patients.

### Relationship between initial ICP and prognosis

aSAH patients often develop intracranial hypertension due to mass effects (hematoma/edema), which may progress to cerebral herniation. Elevated ICP serves not only as a marker of injury severity but also poses life-threatening risks: untreated acute ICP elevation can trigger brainstem compression or global ischemia secondary to critically reduced CPP[14]. It is widely recognized that treating elevated ICP is critical to clinical outcomes [15].

In this study, poor-outcome patients exhibited significantly higher initial ICP (41.33 ± 10.23 vs. 25.89 ± 10.42 mm Hg, p = 0.014), aligning with prior evidence that elevated admission ICP predicts adverse prognosis in aSAH [16]. The normal range of ICP is 5–15 mm Hg, whereas ICP is usually elevated in patients with aSAH due to hemorrhage and secondary injuries (e.g., cerebral vasospasm, cerebral edema, and cerebral herniation, etc.). High ICP leads to a decrease in CPP, and the associated impaired cerebral blood flow may lead to acute brain injury or delayed cerebral ischemia, which may result in unfavorable clinical outcomes for the patient [17], a conclusion that is further validated by the data in this study.

This shows that initial ICP can be used as a rapid predictive parameter of prognosis, and its elevation is significantly associated with poor outcome. This result provides a direct basis for early identification of high-risk patients and optimization of cranial pressure reduction strategies. For patients with high initial ICP, enhanced monitoring and management and individualized interventions are needed to improve the recovery of neurological function.

### Relationship between decompressive craniectomy and prognosis

The Monro-Kellie doctrine postulates that ICP dynamics reflect compensatory volume adjustments among brain tissue, cerebrospinal fluid, and blood within the fixed cranial vault. Increased volume in one component necessitates reciprocal reductions in others to maintain intracranial homeostasis [18]. Following intracranial aneurysm rupture, hematoma-induced compression and inflammatory mediators trigger cerebral edema. When edema surpasses intracranial compliance, elevated ICP ensues. Decompressive craniectomy (DC)—a neurosurgical intervention involving skull removal to accommodate brain swelling—effectively reduces ICP and prevents herniation in refractory cases. While demonstrating neuroprotective effects, DC's impact on long-term functional outcomes remains uncertain.Although there is a lack of evidence for the routine use of DC to treat elevated ICP in patients with aSAH, it remains a last resort after conservative treatment has failed [19]. Therefore, the indications for decompressive craniectomy in patients with aSAH still need to be further explored.

The normal range of ICP is about 5–15 mm Hg, and considering the body's ability to compensate for ICP, we excluded cases with low initial ICP. In patients with initial ICP ≥ 25 mm Hg, DC demonstrated a clinically relevant 4.8-fold increase in favorable prognosis likelihood (RR≈4.8, p = 0.08), albeit below statistical significance. These findings align with guideline recommendations for DC as salvage therapy in refractory intracranial hypertension, despite requiring validation in larger cohorts [20].

After aneurysm surgery, patients may experience fluctuations in ICP due to cerebral edema, cerebral vasospasm, redistribution of cerebral blood flow, and hydrocephalus [21]. Gregory g. Heuer et al. demonstrated that increased ICP is a common phenomenon in patients with aSAH and occurs even in patients with a favorable clinical classification (Hunt-Hess classification) [16]. A meta-analysis on A meta-analysis of intracranial hypertension in patients with aSAH found that increased ICP occurred in up to 70.69% of patients with aSAH, and increased ICP was strongly associated with a poorer prognosis [17]. Before performing craniotomy aneurysm neck clamping or interventional embolization of intracranial aneurysm, lateral ventricle puncture + ICP monitoring probe placement can monitor ICP in real time. Drainage of cerebrospinal fluid can effectively control the ICP, relax the brain tissue, reduce the damage to brain tissue caused by intraoperative pulling and the risk of intraoperative aneurysm re-rupture, and can reduce the irritation to the brain tissue by draining the bloody cerebrospinal fluid in the postoperative period, thus reducing the risk of cerebral vasospasm, as well as reducing the ICP, decreasing the cerebral edema, and improving the cerebral perfusion. In the past, physicians often evaluated the need for decompressive craniectomy based on experience and the degree of brain tissue swelling seen on CT images or with the naked eye during surgery. Intraoperative and postoperative monitoring of ICP can provide physicians with specific data reference to help decide whether to perform decompressive craniectomy. For example, the results in this study showed that at an initial ICP of ≥ 25 mm Hg, decompressive craniectomy may have a favorable impact on the patient's prognosis, which can provide some reference for the operator's decision-making during the operation.

### Relationship between ΔCPP and prognosis

CPP is a key driver in maintaining cerebral perfusion and oxygen supply, and is necessary for brain tissue to receive an adequate blood supply. Traditional guidelines recommend maintaining CPP at 60–70 mm Hg, but this range is mainly based on TBI studies [4]. However, the pathogenesis of aSAH is different from that of TBI and is highly individualized, and a uniform CPP threshold may not be applicable [5]. Several studies have shown that patients with aSAH may benefit from higher CPP thresholds than patients with TBI. For example, it has been shown that keeping the CPP above 70 mm Hg may help prevent delayed cerebral ischemia after aSAH [22]. However, when the CPP is too high, it may in turn induce rebleeding and vasogenic cerebral edema. Therefore, the key to postoperative management of aSAH should be to maintain an appropriate CPP and avoid secondary ischemic or congestive injury as much as possible. The PRx was first proposed by Marek Czosnyka as early as 1997 [6]. Studies have shown that PRx can reflect the autoregulation of cerebral blood vessels, and good cerebral vascular regulation will result in the ability to keep MAP and ICP relatively stable and maintain stable cerebral blood flow (CBF) even when systemic blood pressure fluctuates. This mechanism is believed to play a role in the development of delayed cerebral ischemia [7, 23]. And with the progress of research in recent years, CPPopt has also received widespread attention, which is an individualized optimal CPP value determined based on the dynamics of cerebrovascular autoregulation function, and is defined as the CPP value corresponding to the time when cerebrovascular autoregulation is optimal (when the pressure-responsiveness index PRx is at its lowest). In a small prospective study that included 29 patients with aSAH, a CPP lower than CPPopt was found to be associated with a poorer prognosis [24]. However, other studies have come to the opposite conclusion, concluding that deviation of the actual CPP from the target value of CPPopt derived from the PRx does not correlate with poor outcome [25]. Therefore, the efficacy of individualized CPP management strategies based on CPPopt still needs to be further evaluated, and future studies will help to elucidate the clinical utility of CPPopt.

In this study, we found that the mean ΔCPP of the poor prognosis group was significantly higher than that of the Favorable prognosis group (p = 0.033), suggesting that the greater the deviation of the actual CPP from CPPopt, the worse the prognosis of the patients. This result is consistent with the conclusion of the TBI study that “patients whose actual CPP is close to CPPopt have better prognostic outcomes than patients whose CPP deviates more from CPPopt” [26]. The significant difference between mean ΔCPP smaller than CPPopt and prognostic subgroups (p = 0.038) suggests that hypoperfusion may significantly increase the risk of poor prognosis. The mean ΔCPP greater than CPPopt, although greater in the poor prognosis group than in the Favorable prognosis group (p = 0.084), did not reach significance, with a p-value close to 0.05, suggesting that hyperperfusion may negatively affect prognosis but has not yet been able to be statistically confirmed. Combining these results, we believe that postoperative CPP values that deviate too much from the CPPopt, whether low or high, increase the risk of complications or brain injury in patients, which in turn leads to a poor prognosis. In this case, the negative prognostic impact of hypoperfusion is more significant.

Hypoperfusion usually leads to hypoxia of brain tissue and metabolic restriction of brain cells, which in turn causes cerebral ischemia and metabolic crisis. Due to the higher risk of cerebral vasospasm in patients with aSAH, the effects of hypoperfusion may be more sensitive to its effects. Although the mean ΔCPP greater than CPPopt did not reach a significant difference in the prognostic subgroups, the p-value of 0.084, which is close to 0.05, suggests that hyperperfusion may have a negative impact on prognosis, and in the context of the clinical reality of patients with aSAH, although it has not yet reached a statistically significant difference, which is of interest, as Marcel J.H. Aries et al.'s study in traumatic study in TBI also verified this point [26]. Excessive cerebral perfusion may lead to an increase in cerebral vascular permeability, resulting in the disruption of the blood–brain barrier, which may aggravate the degree of cerebral edema and further increase ICP. Excessive cerebral perfusion may also increase the pressure on the walls of cerebral blood vessels, which may cause rupture of the vessel walls and increase the risk of intracranial rebleeding. The results of this study suggest that individualized management of CPP after aSAH is essential and that postoperative CPP regulation should not depend on uniform fixed criteria. For patients with low perfusion, measures should be taken to improve CPP, such as appropriately raising blood pressure and maintaining appropriate fluid intake; for excessive cerebral perfusion, care needs to be taken to avoid the risks of increased cerebral edema and rebleeding caused by overperfusion.

Although univariate analysis indicated that initial ICP, mean ΔCPP deviating from CPPopt, and mean ΔCPP below CPPopt were significantly correlated with patient prognosis, none of these three variables maintained independent statistical significance (P > 0.05) in the multivariate binary logistic regression model. This finding suggests that complex interactions may exist among these predictors rather than independent prognostic factors. Several potential reasons may account for this.

A key limitation of this study is insufficient sample size, as the prospective cohort included only 15 patients—this may limit the capacity of multivariate models to identify independently significant associations. Although univariate analysis yielded robust signals, multivariate analysis demands larger sample sizes to ensure adequate statistical power for discerning the independent effects of these closely correlated variables. Validation of the independent roles of these variables thus requires future prospective studies with expanded sample sizes.

Another limitation is collinearity among key predictors: patients with high initial ICP have greater difficulty maintaining CPP, leading to larger mean ΔCPP deviations from CPPopt and higher mean ΔCPP values when CPP < CPPopt. When these highly correlated variables are included concurrently in statistical models, the models cannot distinguish their independent contributions, resulting in inflated p-values for each variable—confirming significant statistical collinearity. While multivariate models are designed to isolate the independent effects of individual variables, significant multicollinearity between predictors diminishes the statistical power to detect these individual contributions.

Although not statistically significant, we observed that in the multivariable model, both higher initial ICP and greater ΔCPP had odds ratios (OR) greater than 1 (ICP: P = 0.181, OR = 1.140, 95% CI = 0.941–1.381; mean ΔCPP deviating from CPPopt (P = 0.934, OR = 1.115, 95% CI = 0.086–14.506), and the mean ΔCPP below CPPopt (P = 0.878, OR = 1.231, 95% CI = 0.086–17.537), consistent with univariate analysis results and existing pathophysiological knowledge. This indicates they remain important clinical markers of poor prognosis. In clinical decision-making, particularly when these parameters are markedly abnormal, the risk information provided by their numerical values is highly valuable. The primary limitations of this study include sample size and the aforementioned multicollinearity issues, which may have constrained the efficacy of multivariate analysis. Future studies with larger cohorts are warranted to validate these findings.

### Validity of baseline matching in prospective and retrospective cohorts

In this study, after controlling for key confounders such as age, gender, Hunt-Hess grading, modified Fisher grading, and surgical approach between the prospective and retrospective cohorts by propensity matching (PSM), there was no statistically significant difference in the baseline characteristics of the two groups (all p > 0.05), indicating successful matching. After balancing the baseline confounders, the Favorable prognosis rate was found to be significantly higher in the experimental group that received multiparameter monitoring than in the control group that did not undergo multiparameter monitoring (60.0% vs. 31.1%, p = 0.046), suggesting that ambulatory monitoring based on ICP and individualized CPPopt may improve the outcome of postoperative management in patients with aSAH. This result suggests that the prognostic difference between the prospective cohort (test group) and the retrospective cohort (control group) is more likely to originate from the multiparameter monitoring intervention itself rather than baseline severity of disease or treatment selection bias, enhancing the validity of the findings.

### Clinical significance of prognostic differences and potential mechanisms

Dynamic monitoring of ICP can guide precise treatment. The control group relied only on clinical assessment (e.g., symptom observation, imaging examination) and lacked real-time monitoring of ICP and related parameters, which may delay the recognition of elevated ICP and lead to irreversible damage. The experimental group, on the other hand, through continuous and accurate monitoring of ICP, could recognize intracranial hypertension at an early stage and intervene in a timely manner, adjusting the speed of cerebrospinal fluid drainage, the dosage of dehydrating drugs and the depth of sedation in real time, and reducing the secondary brain damage caused by high ICP.

Postoperative delayed cerebral ischemia (DCI), cerebral vasospasm and rebleeding after aSAH are closely related to poor prognosis, and multiparameter monitoring can provide early warning of complications. In the experimental group, a ventricular ICP monitoring probe was surgically inserted to monitor ICP and related parameters such as CPP and CPPopt in real time during and after surgery, to dynamically adjust the CPP, and to effectively reduce the duration of hypoperfusion (CPP < CPPopt) or hypoperfusion (CPP > CPPopt), thus improving the prognosis of the patients. Part I has demonstrated that ΔCPP (the extent to which actual CPP deviates from CPPopt) is significantly associated with poor prognosis, and that ΔCPP may be lower in the test group, thereby reducing the risk of secondary delayed cerebral ischemia or rebleeding. This mechanism is consistent with findings in TBI studies, where CPPopt-oriented management has been shown to reduce mortality and improve functional prognosis in patients with TBI [26]. The present study validates the effectiveness of this strategy in patients with aSAH, expanding its clinical application scenarios. Through real-time multi-parameter monitoring, it can help clinical treatment decisions, reduce medical wastage and unnecessary treatment, and is expected to help realize a win–win-win situation for patients with improved prognosis, improved quality of care, and sustainable health insurance funds.

### Study limitations

Sample Size and enrollment challenges: Notably, the small cohort sizes (prospective: n = 15; retrospective: n = 45) may lead to overestimation of effect sizes (e.g., the observed 28.9% outcome difference) and restrict subgroup analyses—for instance, the subgroup of patients with ICP ≥ 25 mm Hg (n = 11) lacks sufficient power for meaningful inference. Additionally, enrollment challenges—partly attributed to economic constraints and the relative novelty of ICP/CPPopt monitoring in institutional practice—further limit the external validity of the study findings.

Methodological Biases: This study is subject to several methodological biases. First, retrospective control data may contain unmeasured confounders (e.g., inconsistencies in treatment protocols), which could skew outcome associations. Second, non-random allocation of decompressive craniectomy introduces selection bias, as decisions were based on clinician judgment and patient-specific factors rather than a randomized protocol. Additionally, the study sample size was not determined via formal power calculations, increasing the risk of Type II error (i.e., failure to detect a true difference). Consequently, this research should be categorized as exploratory/hypothesis-generating; its findings merely indicate an effect trend, which requires further validation in larger cohorts with pre-calculated statistical power.

Analytical Constraints: Exclusive focus on ΔCPP-prognosis correlations precluded multivariate adjustment for competing risk factors, potentially obscuring confounding effects. Additionally, lack of long-term outcome data restricts insights into the sustained clinical impact of studied variables: the relatively short follow-up period (up to 1 months postoperatively) – during which most patients had achieved favorable recovery (GOS 4–5) – meant long-term functional outcome data (e.g., 6-month or 1-year GOS scores) were not collected. This limits our capacity to evaluate factors affecting long-term neurological outcomes. Future prospective long-term follow-up studies are planned to address this limitation.

Unmeasured confounding factors: Despite all surgeries being performed by or supervised by qualified neurosurgeons at our institution and adhering to standard treatment protocols, we were unable to quantify or statistically adjust for inter-surgeon experience variability—this may constitute an unmeasured confounding factor. Additionally, other difficult-to-quantify variables (e.g., patients’ genetic background, socioeconomic status, and post-discharge rehabilitation intensity/quality) that could influence prognosis were not assessed in this study.

Study Design and Monitoring Accessibility: The single-center design introduces inherent institutional bias, necessitating multicenter validation to enhance the generalizability of findings. Additionally, the invasive and costly nature of multimodal monitoring impedes its widespread clinical implementation, limiting broader adoption of the proposed management approach. Future Directions: Expand cohorts to validate initial ICP thresholds and CPPopt-guided protocols.Integrate multivariate regression and long-term follow-up. Standardize multimodal monitoring protocols for cost-effective implementation.

## Conclusions

This study focused on ICP monitoring and prognosis-related factors in patients with aSAH, and the key conclusions are as follows:​

First, in the clinical management of aSAH patients, ICP monitoring plays a pivotal role: it not only facilitates surgical decision-making (e.g., selection of decompressive craniectomy) but also optimizes postoperative hemodynamic management strategies tailored to individual patients. Second, two key factors were identified to predict poor prognosis in aSAH patients: one is the deviation from postoperative CPPopt, among which hypoperfusion is a particularly impactful subtype; the other is elevated initial ICP. Furthermore, this study confirmed that management guided by multiparameter monitoring significantly improves short-term clinical outcomes in aSAH patients, providing practical evidence for the optimized clinical management of this patient population.

## Data Availability

No datasets were generated or analysed during the current study.
